# Public involvement in research about environmental change and health: A case study

**DOI:** 10.1177/1363459318809405

**Published:** 2019-02-20

**Authors:** Kath Maguire, Ruth Garside, Jo Poland, Lora E Fleming, Ian Alcock, Tim Taylor, Helen Macintyre, Gianni Lo Iacono, Andrew Green, Benedict W Wheeler

**Affiliations:** University of Exeter, UK; Health and Environment Public Engagement (HEPE), UK; University of Exeter, UK; Public Health England, UK; University of Surrey, UK; Health and Environment Public Engagement (HEPE), UK; University of Exeter, UK

**Keywords:** environment and health, health policy, issues in research methodology, theory

## Abstract

Involving and engaging the public are crucial for effective prioritisation, dissemination and implementation of research about the complex interactions between environments and health. Involvement is also important to funders and policy makers who often see it as vital for building trust and justifying the investment of public money. In public health research, ‘the public’ can seem an amorphous target for researchers to engage with, and the short-term nature of research projects can be a challenge. Technocratic and pedagogical approaches have frequently met with resistance, so public involvement needs to be seen in the context of a history which includes contested truths, power inequalities and political activism. It is therefore vital for researchers and policy makers, as well as public contributors, to share best practice and to explore the challenges encountered in public involvement and engagement. This article presents a theoretically informed case study of the contributions made by the Health and Environment Public Engagement Group to the work of the National Institute for Health Research (NIHR) Health Protection Research Unit in Environmental Change and Health (HPRU-ECH). We describe how Health and Environment Public Engagement Group has provided researchers in the HPRU-ECH with a vehicle to support access to public views on multiple aspects of the research work across three workshops, discussion of ongoing research issues at meetings and supporting dissemination to local government partners, as well as public representation on the HPRU-ECH Advisory Board. We conclude that institutional support for standing public involvement groups can provide conduits for connecting public with policy makers and academic institutions. This can enable public involvement and engagement, which would be difficult, if not impossible, to achieve in individual short-term and unconnected research projects.

## Introduction

Institutions and funders increasingly require demonstrable public involvement in public health research, implementation and practice, not just in health services research. This can be difficult for researchers to implement in individual projects (Brunton et al., 2017), particularly if these are policy-driven, as projects about interactions between environments and health often are, because policy-driven projects tend to be short term and immediate. Public engagement and involvement can then seem excessively time-consuming. In addition, while more clinically focused health service researchers are often able to identify particular patient or service user groups who they see as obvious candidates for involvement in their work, researchers looking at the complex interactions between environments and public health concerns can find it difficult to conceptualise the public or ‘publics’ with whom they need to engage. Furthermore, where very broad and universal topics are under consideration, researchers may be anxious about a perceived need to involve a statistically representative public group, generally an unrealistic aspiration which can preclude achievable involvement activities and any benefits these could deliver (Maguire and Britten, 2017).

Proposals to develop functional plans or policies need to be informed by the views and experiences people who will use them or benefit from them. The ultimate goal of research about the interactions between environment and public health is to produce knowledge which can be effectively translated into public policy and which can influence systems, organisations and individual lifestyle choices. There have been many successful public health programmes including the impact of the UK Clean Air Acts of 1956, 1968 and 1993; yet, there is also a long history of public resistance to public health, and perhaps particularly environmental interventions, when implemented in ways perceived as top down, technocratic and pedagogical. Examples range from the cholera riots of the early 19th century (Burrell and Gill, 2005) to current campaigns against fluoridation and pollution control. ‘Forced-fluoridation Freedom Fighters^1^’; and ‘Coal Rolling’ (Schlenger, 2014; Tabuchi, 2016). It is important to recognise that research about relationships between the environment and public health takes place in a context which includes contested truths, political activism and power inequality (Contandriopoulos, 2004).

The way health issues are reported in the popular press has sometimes added to public confusion and doubt. For instance, two headlines in the same newspaper just over a month apart read ‘Bowel and gullet cancer: Just two beers or glasses of wine “raises your risk”’ and ‘Wine is KEY to a longer life: Daily drink can slash the risk of an early death’ (Daily Express, 2017; Reynolds, 2017). Newspaper coverage of ‘superfoods’ has also raised concerns that the promotion of research may sometimes be overly influenced by commercial concerns (Weitkamp and Eidsvaag, 2014). These examples demonstrate that media messages about health risks and benefits which confront people when making day-to-day choices are often confusing or misleading.

These issues have led institutions to identify a perceived public deficit in scientific understanding, but it has also been argued that the culture of science-policy institutions could be a contributory cause of public mistrust (Wynne, 2006). While five types of barriers to the effective involvement of communities by statutory organisations were identified by Picking et al. (2002), only one of these refers to a potential lack of capacity to engage on the part of the community. The other four barriers identified were a lack of organisational staff skills and competencies; dominance of professional cultures; unsupportive organisational ethos; and local or national political dynamics. As research about the environment and public health increasingly consists of multi-sector collaborations which may include statutory, academic and private sector partners, some of these institutional issues may become amplified within individual research programmes and projects.

Drivers of public involvement in environmental and public health interventions can be broadly categorised as based on either a ‘utilitarian’ perspective (i.e. focused on achieving specific health, informational or service delivery outcomes) or as motivated by ‘social justice’ and the redistribution of power and knowledge (Brunton et al., 2017). Those responding to these drivers have been characterised as either ‘pragmatists’ or ‘activists’ (Morgan, 2001). These categories can serve as a useful heuristic, to encourage thought and discussion about the aims and hoped for outcomes of public involvement processes when planning a specific research project or programme. In practice, however, there are likely to be a range of internal and external influences on any particular public involvement activities, introduced through differing individual, organisational and community interests (Oliver et al., 2015). It is also possible to imagine a middle ground where these perspectives converge, for instance, if it were believed that improved health outcomes follow from the more effective use of information and resources by empowered individuals and communities, or that support for the public funding of research is promoted by the democratisation of knowledge creation.

There are different definitions of public involvement and engagement utilised in this context. For example, the Research Councils UK (RCUK, 2014) uses ‘Public Engagement’ as an overarching term for activities in which the public are provided with access to knowledge generated through research and/or opportunities to influence research agendas. However, the National Institute for Health Research (NIHR) INVOLVE makes clear distinctions between (a) *involvement*: the active involvement of the public in projects or organisations; (b) *engagement*: the provision of information and knowledge about research; and (c) *participation*: where people are recruited to take part in research, including trials, focus groups or completing questionnaires (INVOLVE, 2018a).

Many of the activities classed as ‘public engagement’ by the NIHR INVOLVE definition may be external to particular research projects, or something which follows from their production of knowledge. Examples include presenting at public science fairs; participating in popular media programmes; holding institutional open days or public debates; and disseminating findings in lay terms to research participants and wider public interest groups. However, ‘public involvement’ implies a much closer working relationship and the ceding of some control to the involved public. Examples of public involvement include acting as advisors or steering group members, taking part in research prioritisation, advising on or co-producing the content and presentation of questionnaires or research documents, informing the structure of mathematical models, gathering data, contributing to data analysis and disseminating findings with or for the research team. Thus, the role of members of the public in public involvement is as advisors, co-applicants and co-authors and co-creators; colleagues rather than subjects, clients or audiences.

One way of conceptualising the complex range of activities that make up public involvement, drawing on sociological literature, is to see it as creating ‘knowledge spaces’ (Elliott and Williams, 2008; Gibson et al., 2012). This is a metaphor for structures which bring together people with different sorts of knowledge, understandings and experiences of a topic or phenomenon. Within these spaces, people act as co-contributors to what Jasanoff (2000) described as ‘civic epistemology’, processes which evaluate and utilise knowledge in societal decision-making.

In the context of research about the environment and public health, public involvement knowledge spaces bring together people who may approach issues from different perspectives: individual personal and relational, rational scientific, and often also political and/or public policy. This means these types of spaces are seen as existing somewhere in between the more formal institutional spaces and everyday informal relationships (Maguire and Britten, 2018). This liminal quality of knowledge spaces helps to explain why they can be difficult to establish by individual researchers running short-term projects. It takes significant investment of time and effort to build contacts, trust and ways of working together. In addition, the drivers of public involvement are often experienced at the local level, rooted in place, with communities helping shape the factors impacting on their health, and frequently involving a range of voluntary and community sector organisations. These drivers have led a number of research institutions to found standing public involvement groups.

This article uses our theoretical understanding and practical experience of public involvement in the work of The European Centre for Environment and Human Health (the Centre) to explore challenges and opportunities for creating a flexible knowledge space to enable effective involvement in public health research. In particular, we use the example of involving people in a specific set of our research activities, with the NIHR Health Protection Research Unit in Environmental Change and Health (HPRU-ECH), as a case study to demonstrate the added value brought to the Centre by our standing public engagement group the Health and Environment Public Engagement Group (HEPE).

## Context

The Centre is part of the University of Exeter College of Medical and Health, based at the Truro campus in Cornwall (ECEHH, 2018). Our research encompasses both emerging threats to health and well-being posed by environmental change, and the health and well-being benefits the natural environment can provide. This has included a broad range of research topics including the risk of infection from seawater (Leonard et al., 2018); relationships between pro-environmental attitudes and household behaviours (Alcock et al., 2017); health and well-being benefits of biodiverse environments (Lovell et al., 2014); and the relationship between coastal proximity and physical activity (White et al., 2014). This has led us to develop a truly inter-disciplinary team that crosses traditional disciplinary boundaries to include epidemiology, sociology, geography, policy analysis, systematic reviews, health economics, psychology, anthropology and microbiology (Phoenix et al., 2013).

The Centre was launched in 2011 with support from Convergence, the European economic regeneration programme for Cornwall and the Isles of Scilly. So, from the outset, the Centre has aimed to produce research that contributes to social and economic well-being in the South West England, as well as having impacts on national and international policy. This has led to the development of ongoing and close working relationships with local government, businesses and voluntary organisations, as well as with a range of national and international research partners.

In 2013, R.G. secured seed funding from the RCUK Catalyst project^2^ at University of Exeter to found a standing group of local people to work with the Centre. Practical support for this was provided by the expert Patient and Public Involvement team from NIHR Collaboration for Leadership in Applied Health Research and Care, Southwest Peninsula (PenCLAHRC).^3^ Invitations to introductory information events at the Centre were circulated through local radio, social media and through direct contact with community and environmental interest groups from across Cornwall.

From these events a standing group of 12 people were recruited and they chose the name ‘Health and Environment Public Engagement’ (HEPE).^4^ The intention was for the group to act as interested individuals and critical friends to researchers working in the Centre. They were not intended to act as representatives of any particular group or community. Over time, the interests that have led people to join HEPE vary a great deal. They include public access to academic institutions; environmental sustainability; telehealth and low-carbon futures; links between food and mental health; and collaborating with others to find out more about the interaction between environment and health:

I love being part of the HEPE group. I feel that our time and opinions are truly valued and taken account of. It’s good to meet the people who are doing the research and feel able to help them. My fellow HEPE colleagues have also enriched the work I do as a Volunteer Coordinator for Cornwall Butterfly Conservation. (J.P. HEPE member)

Since the autumn of 2013, HEPE has met quarterly. There have also been additional workshops and email consultations about specific research projects between meetings. Our policy, based on those of the PenCLAHRC team, is to reimburse members’ travel costs in cash on the day and to offer a small honorarium in recognition of their contribution, paid into their bank account. The HEPE mailing list is reviewed annually, and any member who has not been in contact for more than 6 months is contacted to check whether they wish to continue their involvement. New members are recruited through outreach events and activities as well as by word of mouth and social media.

At the time of writing, six of the original HEPE members remain among the 25 people on our current mailing list, and through HEPE, more than 60 individuals have contributed to over 40 research projects in the intervening 4 years. HEPE members have contributed across the broad spectrum of research taking place in the Centre including work exploring human exposure to antibiotic resistant bacteria in coastal waters (Leonard et al., 2015); older people’s sensory experience of the natural world (Orr et al., 2016); and potential benefits to public health and well-being from Europe’s blue spaces (Grellier et al., 2017). These contributions have ranged from prioritising research questions and assessing and revising new plans for research to providing feedback on questionnaires, lay summaries and presentations which communicate research findings. HEPE members have also presented on public involvement at conferences and contributed to teaching at undergraduate and post-graduate levels.

This work has been sustained through the commitment of Centre staff with continuing support, in terms of both funding and staff time, from PenCLAHRC, as well as from sponsorship of individual workshops and activities from the budgets of specific projects which have used the resources of the group. R.G. has also received personal funding for work with HEPE as a RCUK Catalyst Public Engagement Champion. As well as involving HEPE in research projects identified by researchers, the Centre has also accessed funding to pursue interests arising within the group, provided training and supported networking opportunities.

## Health Protection Research Unit in Environmental Change and Health

In 2014, NIHR founded 11 Health Protection Research Units (HPRUs) as partnerships between academic institutions and Public Health England (PHE). These are intended to act as multi-disciplinary centres of excellence for health protection research.

The HPRU-ECH^5^ is a partnership between the London School of Hygiene and Tropical Medicine (LSHTM), PHE, the University of Exeter, the Met Office and University College London (UCL). It focuses on the health and well-being impacts of climate and other environmental change, and how these can be responded to by local, regional and national public health decision-makers. Research within this group aims to develop knowledge and tools that can facilitate adaptation and interventions which can mitigate negative impacts, and also promote benefits from changes in climate, land use and ecosystem services. These activities are intended to support PHE and other government bodies in developing and fulfilling public health policy requirements on adaptation to climate and other environmental change and on environmentally sustainable development.

The research of this partnership is organised in three interconnected themes: (1) Climate Resilience, led by PHE and the LSHTM; (2) Healthy Sustainable Cities, led by PHE and UCL; and (3) Public Health and the Natural Environment, led by PHE and the University of Exeter at the Centre for the Environment and Human Health. It is this third theme in which HEPE has been most closely involved. This theme explores the role of green/blue space (areas of vegetation and/or water) and the natural environment in improving mental and physical health by linking relevant information from large data sets, as well as by exploring the effects of environmental change (including changes in climate and land use) on the transmission of infectious and vector-borne diseases, and on non-communicable diseases through changes in aero-allergens (such as pollen and harmful algal blooms).

## Design and conduct of involvement

Once the HPRU-ECH partnership was established, L.E.F., director of the Centre, attended a HEPE meeting where she described the research priorities that had been set by the funders and the structure of the partnership. The group then discussed what aspects of the research they found particularly interesting and how they could most effectively influence the research agenda. The health impacts of access to the natural environment is a subject that HEPE had a long-standing interest in pursuing. An issue that interested the group was uncertainty about the exact questions which would be used to interrogate large data sets in order to explore the interaction between individual’s access to green/blue space within their local environment and their health. So, it was agreed to run annual workshops with Centre researchers working on the HPRU-ECH, to explore these issues and questions that arise from them. In between these workshops, HEPE would be kept informed of the work of the HPRU-ECH, and the group also offered to provide representatives for the HPRU-ECH Advisory Board.

Because this is public involvement in which members of the public are acting as special advisors and activity is constituted of consultation, collaboration and co-production of the research, as opposed to data gathering, these activities did not require review by a research ethics committee (INVOLVE, 2018b).

### The first workshop – 2015

The first workshop took place in November 2015. The HPRU-ECH research team from the Centre worked with K.M., an expert in public involvement from PenCLAHRC. They discussed the requirements outlined in the HPRU-ECH funding agreement, and identified uncertainties and issues which needed to be prioritised in order to plan their ongoing research programme about the health and well-being implications of access to green and blue space in the local environment. From this, they developed a workshop plan which included a series of structured activities to enable focused discussions on these topics with members of the public.

Six members of the research team and fourteen members of the public attended the workshop (six of these were HEPE members and eight were from their wider networks including community, environment and wildlife groups). Information about the national HPRU programme, the remit of the HPRU-ECH and the role of the Centre were circulated in advance.

The workshop began with an icebreaker exercise where people were invited to distribute ‘coin’ stickers between public health and clinical services. This was intended to get people mingling, to open discussions about their own priorities for health funding in general and to explore the distinction between public health initiatives and clinical services.

The second activity was a series of small group discussions about the impact that perceptions of environmental risk have on access to nature. People moved between tables each with a researcher facilitating discussion of a slightly different question. These explored what information sources people access; how this influences their access green space in the local environment; and how information could be improved. Everyone was encouraged to jot their ideas onto the tablecloths. There is not space here to present all these notes, but an area of broad agreement was the prioritisation of information on how to mitigate risks rather than simply identifying them. For example, too many ‘danger’ signs were seen as making the outdoors seem intrinsically risky, leading people to greater health risks through avoidance of these spaces and resultant inactivity.

The research team then presented a brief explanation of the data resources they intended to access in their upcoming work on how access green space in the local environment might be related to health. They delivered a series of individual ‘pitches’ for particular health research issues which could be prioritised for investigation using that data. These included Physical Activity; Obesity; Type 2 Diabetes; Respiratory Health; Mental Health; Social Relations; and Costs of Disease and Cost-benefit Analysis. In an initial vote, taken immediately following the pitches, Mental Health gained the most votes with Physical Activity and Social Relations coming in at joint second. There were then roundtable discussions about why these decisions had been made, followed by a second vote. In this second round, Mental Health still came out top, but Physical Activity was pushed in to third place by Social Relations.

Interestingly, discussions and comments posted on the board suggested that people had not voted for Costs of Disease and Cost-benefit Analysis because they believed that these should be an integral part of any study, rather than a topic in themselves. This is a good example of why it is important to have multiple ways of capturing information from workshop activities had we only recorded the votes rather than the discussions that underpinned them, we might not have recognised that Costs of Disease and Cost-benefit Analysis was seen as a high priority.

Following this event (and also following all subsequent events), feedback was circulated to the workshop attendees, other HEPE members and the researcher partners in the HPRU-ECH. The priorities identified in the workshop became a framework for planning research activities within Theme 3 of the HPRU-ECH.

### Second workshop – 2016

A year later, HEPE members and people who had taken part in the first workshop were invited to a second event. This was intended to give feedback on the progress of the research and to enable discussion of issues that had arisen since the last meeting. Nine members of the public were able to attend. Seven were HEPE members, two of whom had joined the group after attending the first HPRU-ECH workshop. Three members of the public involved in this workshop had not attended the previous year. Five members of the research team also took part.

The workshop began with a presentation about the work which had already taken place. The research team were initially concerned that their progress fell short of what would be expected, particularly on the prioritised issues of the impacts of access to green space in local environments on Mental Health and Social Relations. This was because the team had needed to address a number of time-consuming administrative and data security issues in order to link the different data sets they wanted to work with. In the event, the group were very supportive and actually welcomed the fact that data security and safeguarding of personal privacy were being taken so seriously by the team.

The bulk of the workshop was devoted to an exercise intended to take forward discussions from the 2015 meeting, about how to use the concepts of cost-effectiveness and value for money in the context of health impacts of access to green space in local environments. This involved discussion of how utility values are commonly calculated. The group were then introduced to the items covered by the General Health Questionnaire (GHQ-12; GL Assessment, 2017) and the 12-Item Short Form Health Survey (SF-12; Optium, 2018), as data gathered using both of these were available in the data sets being accessed.

Three groups, each including a researcher and three public contributors, were given bullet point lists of symptoms experienced by a fictional individual before and after an imagined ‘green space’ intervention. The interventions included regular organised bus trips to public parks or gardens; a scheme for sharing gardening skills with allotment holders and school students; and volunteering on a tree planting project. The group were asked to discuss whether they felt, based on these ‘symptoms’, the intervention had made a difference to the individual. If so, was this a difference likely to be important to that individual and would this be identified by their responses to the GHQ-12 and the SF-12 Health Survey?

The groups were then given a narrative about the same individuals, giving more of their personal and social background, and how the green space intervention interacted with other aspects of their lives. Workshop contributors were asked to review their previous answers in light of these insights. Researchers reported that these discussions were extremely helpful in highlighting the complexity of links between green space interventions, well-being and health. In particular, the workshop raised the magnified importance of what might appear to be quite small differences for people with multiple physical and mental health as well as social issues. Researchers were particularly interested in the scepticism public contributors voiced about the potential for these positive impacts to be identifiable in the data. Many of the group’s comments echoed the prioritisation of Mental Health and Social Relations which had been identified by attendees at the previous workshop.

Discussions arising from this event were particularly influential in terms of how members of the research team subsequently approached the interrogation and interpretation of data.

### Third workshop – 2017

The third workshop took place in November 2017. It involved 10 members of the public – 4 of whom had not attended either of the previous workshops and 2 who were not HEPE members. Five members of the research team also attended. Information from previous workshops was circulated in advance.

The first part of the workshop again provided an update on the Centre’s research within the HPRU-ECH. The research team presented ongoing work on links between the natural environment and physical and mental health as well as work comparing the health and well-being impacts of exposure to nature with those of social connectedness.

The design of research into nature exposure and Social Relations, a topic which had been highlighted as an important issue in the two previous workshops, became the main focus of the afternoon. I.A. proposed to explore associations between residential area natural environments and the quality of people’s relationships with their spouse/partner using archived data available from the UK Household Longitudinal Survey (UKHLS, Understanding Society^6^) as a possible measure of Social Relations. These questions ask about how partners communicate; shared activities; and whether they regretted or thought about ending the relationship.

The proposed research would examine statistical relationships between the individual UKHLS participant scores on the measures to be developed from responses to a multi-item questionnaire about people’s marital relationships and their residential area exposure to natural environments. This could help understanding about whether health is improved directly by exposure to natural environments, or whether this occurs indirectly because these environments promote better social relationships.

The objective of the workshop was to explore views about which, if any, of the UKHLS questions on partner relationships were most relevant to measure of the quality of these relationships. First, we discussed that, in this context, for a measure to be ‘valid’ in terms of the proposed research, it should be equally useful for all social groups; and for it to be considered ‘relevant’, it should really measure the quality of partner relationships, and not something else like the different cultures, habits or expectations of particular groups or social classes.

We again used one of the narrative scenarios from the previous workshop, mapping an individual’s social relations before and after a green space intervention. The scenario provided a focus for discussions about how useful a range of UKHLS questions would be in identifying differences in the quality of these relationships. Another aspect under discussion at the workshop was whether and how exposure to green space might affect an individual’s answers to these questions.

While some of the questions seemed relevant, concerns were expressed about how the language used in others might be understood differently by different groups. For example, the idea of ‘calmly discussing’ was not seen as something which would be particularly valued by all groups or cultures, for some people having more excitable conversations might not show anything negative about their partner relationships. Similarly, whether people had thought about divorce was seen as something likely to be heavily influenced by cultural and religious beliefs.

It was also felt that questions about whether partners’ shared interests could be misleading as often having diverse interests is part of a good relationship. Yet, the group believed there could be a plausible connection between the quality of individual personal relationships and green space exposure, suggesting that going for a walk together in the countryside or park, for example, might immediately lead to people exchanging ideas, as being on the walk might give them the chance to discuss things, right then and there.

## Ups and downs

We judge the involvement of HEPE in the HPRU-ECH to have been successful and impactful: it has helped to shape and prioritise the research agenda, provided insights which supported analysis and contributed to presentations. Yet, we would not want to paint it as having been without difficulties and misunderstandings. Not all the research team have been enthused and confident throughout, and not everything public contributors have wanted the project to achieve has been possible.

It has been challenging for some members of the research team to involve themselves in arenas that expose their scientific rigour to potential criticism from the perspective of ‘lay’ (perhaps here best understood as ‘unsanctified’ knowledge; Britten and Maguire, 2016). Also, as mentioned above, the requirement to return to the group a year after asking for their research priorities and admit that little progress had been made on some of these caused a degree of trepidation. In both cases, these concerns were successfully addressed through engaging in ways that were honest and focused. Open discussions have built trust and supported positive future engagement.

Not everyone who has taken part from a public perspective has been able to recognise the requirements of the research, sometimes seeking to raise issues which could not be addressed effectively in that arena. Having a place where personally important but currently tangential issues can be collected and reported in feedback has proved helpful. Sometimes, it has been possible to identify other research where these issues are relevant so we can signpost people to those projects or organisations. Sharing the agenda in advance, shaped around structured activities, has also helped people to remain focused. These are tools that group members use to remind each other why they are there, taking some control of the space and keeping the discussions on track for themselves.

Sometimes, HEPE members have been critical of the support they have received. For example, after a separate dissemination workshop we ran, which was largely aimed at local government employees with responsibilities for managing public open spaces, we received feedback from public contributors who expressed dissatisfaction with the level of briefing we had provided before the event. They felt their role had not been sufficiently clear, so they were left feeling uncertain about how valuable and impactful their presence at the event had been. This feedback was discussed by the specific research team, and lessons were drawn from it to inform similar work in the future, deciding to offer a more detailed face-to-face pre-event briefing as well as sending information by email and/or post.

Public contributors, and indeed researchers, have sometimes been frustrated by institutional barriers and delays which impact on engagement. For example, when L.E.F. first discussed the HPRU-ECH’s work with HEPE, the group proposed having two open seats on the Advisory Board, rather than nominating a representative. This would have given the group flexibility and ensure representation was not dependent on the availability of an individual member. It would also have been an opportunity for members who had not previously been involved in this sort of work to be mentored by more experienced members. In the event, due to organisational and budgetary issues, they were only invited to send a single nominee to attend two meetings. The group has seen this as an opportunity missed.

The experience of involving the public in the HPRU-ECH has been described by researchers as ‘playing catch-up’, because funder’s expectations and definitions of public involvement were not clearly understood by the whole team at the outset. HEPE members have suggested that funders of public health research could be more proactive in communicating information about public involvement and ensuring that realistic public involvement plans are in place before they award major grants. This would require accessible public involvement infrastructures and resources which could support effective planning for involvement at an early stage, as well as infrastructures able to include public contributors in these planning processes. Better reporting of public involvement activities and costs as well as involving the public more effectively in project monitoring and reporting have also been raised by HEPE members as drivers towards more embedded and effective involvement practices.

## Discussion

Although the HPRU-ECH workshops have involved 20 different members of the public over 3 years, the continuity of engagement has been maintained through the distribution of electronic feedback to HEPE and discussions at their quarterly meetings. This has enabled contributions from many more people unable to attend workshops, thus broadening accessibility and supporting ongoing interest in the project; leading us to argue that the value provided to the research and the quality of the discussions taking place were not just a product of the workshops themselves. The regular cycle of HEPE meetings and the resultant creative and participatory information sharing have supported the creation of an open knowledge space connecting the Centre and the wider community.

This has been about building relationships over the long term which, as described above, is not to say that everything always runs smoothly. As in any relationship, there have been some disagreements and some difficult discussions. There would be little point in engaging if we always agreed, and it is important for ‘critical friends’ to be able to maintain that critical edge. The point is for us to be able to communicate disagreements, find a way of understanding our differences and move beyond them. To achieve this, researchers and members of the public need supported spaces to build their confidence and skills to engage effectively. A standing group like HEPE can act as a vehicle to enable this, but it can only be achieved through the investment of time and resources by funders and research institutions.

Research cycles do not run smoothly or evenly. Because of the way that funding rounds, ethics committee meetings and other researcher deadlines fall, there are times when there are many opportunities to be involved and other times when these opportunities seem scarce. A standing group can share out tasks when researchers are being demanding, particularly if people are offered different ways of contributing tailored to their existing skills and availability. Supporting the group to pursue their own interests, sharing information, skills and networking opportunities can help to fill the gaps. This sort of reciprocity helps to sustain relationships with, and within, the group (Mathie et al., 2018), as well as providing the welcoming research environment and support for group members as research partners which has been identified as vital in the context of patient/carer involvement in research (Black et al., 2018).

**Figure 1.**
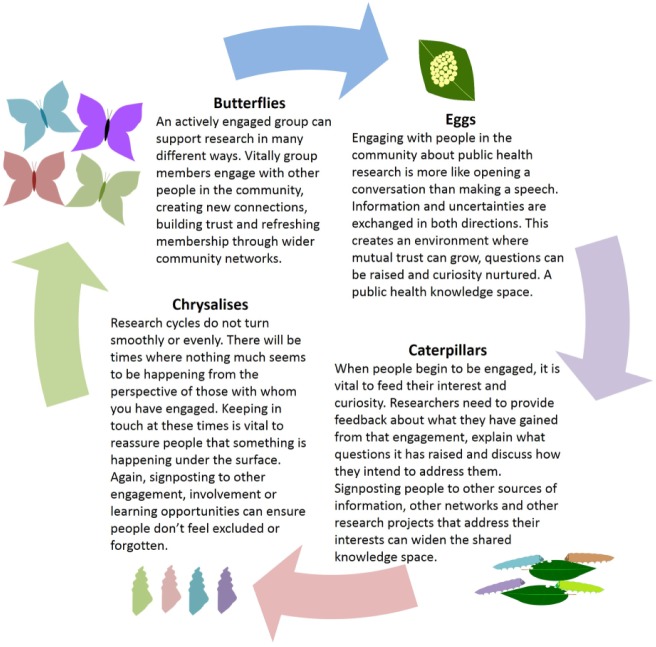
Engagement knowledge space: a conceptual model with butterflies.

Working with J.P., a member of HEPE who also works as a volunteer co-ordinator for Butterfly Conservation, we have developed a conceptual model for the cycle of public engagement, with butterflies (Figure 1). It is intended to demonstrate that, provided with sufficient resources, engagement is an ongoing process which has the capacity to be self-renewing and productive. But this is not a trivial process, it is about changing the relationship between the public, public policy and academic science in order to enable the effective mobilisation of knowledge in multiple directions, and for the benefit of us all.

The sustained effort required is not efficiently supported by short-term grant funding; in this context, 3–5 years is still short term. In order to enable effective public involvement and engagement in public health research, funders and research institutions need to provide core institutional support for open-ended public involvement knowledge spaces. This means tackling institutional inertia, investing in skills and resources for ongoing public engagement and enabling cultures of publicly engaged research (Hinchliffe et al., 2018). As Pickin et al. (2002) has argued, developing a more participatory culture means questioning and re-evaluating dominant professional values; it also requires organisations to develop structures that support openness rather than risk-aversion. In order to achieve this, we need to ensure that we share our experiences of involvement and engagement, the activities we undertake, their impacts on our research and the difficulties we encounter in the process (Staley, 2015).

Involvement of the public in research is still facing many challenges. In the context of rural settings, Chambers (1983) argued that local people are often considered insufficiently knowledgeable to engage in scientific debates on the issues that might affect them. This statement is probably true of the way other populations are viewed, for example, people in other socio-economic situations, especially when the scientific debate involves ‘hard science’ such as mathematics. We argue that integration of participatory research with research methods such as traditional mathematical modelling can be highly beneficial in public health research. For example, the deliberative involvement of the public, patients and carers can help to shape the structure of mathematical models, and/or selection of the most relevant parameters of the system (Grant et al., 2016; Scoones et al., 2017). Groups like HEPE can be involved in identifying desirable outputs from mathematical and other ‘hard science’ approaches, in assessing the assumptions often made in models and hypotheses, in critically reviewing the findings, in designing and evaluating software and web-based implementation of theoretical approaches, in producing lay language versions of scientific papers and in dissemination of research findings.

## Conclusion

The development of productive public involvement in the work of HPRU-ECH’s Theme 3 has been facilitated and sustained by the Centre’s existing and continuing relationship with HEPE. In this, HEPE has acted as more than a standing group of critical friends, it has become an increasingly powerful, skilled and trusted, open-ended knowledge space; connecting researchers and students with their local communities. This has not been about creating a sounding board which will echo and broadcast institutional views. It is a space in which differing ideas and experiences can be exchanged and explored. Yet, it is important to recognise the constraints acting upon this.

The physicality of ‘knowledge space’ as a metaphor implies a position in relation to other spaces; this led Massey (1992) to argued that it is important for such spaces to be explicitly located in their broader social and political landscape. A location which has been proposed for an ideal public knowledge space in health research (Maguire and Britten, 2018) is one equipoised between what Habermas (1984, 1987) describes as the ‘system’ of rational scientific thinking (inherent in institutions) and the ‘lifeworld’ of relational values (associated with personal and familial relationships). Such a space would be equally owned by those taking part in it and would embody the criteria of the ‘ideal speech situation’ (Habermas, 2001) by including anyone able to make a relevant contribution; ensuring that each has equal opportunity to speak; and guaranteeing the absence of all deception or coercion.

HEPE does not achieve that ideal. The group was instigated by researchers in a university. It is resourced in terms of funding, meeting facilities and project work, through those researchers and that institution. Those resources are limited and only accessible to the group through the research team. This dependence can be seen as entrenching an already inequitable relationship between HEPE members and researchers, given that cultural and social capital is inherent in academic status. But these are not the only inequalities acting within this space. Differences of power and status within organisations introduced by career and management structures can constrain candour (O’Toole and Bennis, 2009), and this could inhibit the openness of researchers among themselves and with HEPE; the ambition to enable a socially and culturally diverse membership introduces potential inequalities between HEPE members in terms of race, class, gender, educational attainment and so on.

These issues imply that encounters within this space will always fall short of an ‘ideal speech situation’. In practice, it may be more useful to see those criteria as aspirational goals rather than requirements (Gillespie et al., 2014). There is value in recognising that, within our limited resources and capacities, we cannot fully achieve equity. As Nancy Fraser (1990) has argued, ‘participatory parity’, a situation where ‘ideal speech’ criteria are met, is further undermined if we fail to remain attentive to existing inequalities. It is vital for us to identify those issues which arise from differences in power, capacity, culture, resources and skills between contributors in order for us to act in a way that addresses and mitigates them. What we have been able to create is a flexible vehicle for involvement which provides people with opportunities to contribute in different ways and at different times. Within this, the use of different skill sets and cultural resources are valued. Support and training are also available where there is an appetite for these. The playing field is still uneven, but recognising this enables some mitigation of inequalities.

Mitigating inequalities is and will remain an ongoing task of engagement; it is a process of identifying differences, developing understandings and building relationships. In this article, we have outlined in detail how we have approached this by designing a range of activities that support members of the public to engage with the research of the HPRU-ECH and the broader work of the Centre. These activities support researchers to engage with people who view their work from different perspectives. This is not a matter of diluting the quality of research or undermining the skills brought to bear on research questions. Engaging with the public adds to the knowledge informing the research and ensures its findings are more effectively communicated to those able to apply them in practice. Interdisciplinary research into the interactions between environments and human health is a particularly interesting field to develop this type of publicly engaged research. Our work on the HPRU-ECH already combines different types of knowledge, understanding, skills and methodologies; deals with complex and interacting systems; and requires translations between distinct discourses. In addition, research about environmental change and health encompasses a range of topics which are often of immediate personal interest to members of the public.

This provides us with an opportunity to build strong relationships between research institutions and communities, which we hope will act as robust and ongoing connective knowledge spaces. Members of the public, researchers and policy makers will arrive with different agendas, skills and requirements. No single knowledge space will be able to contain all of the things people bring to them or require of them; this means it is important to support networking and signposting to other opportunities and resources. Ongoing structural support, resources and facilitation are needed in order to sustain engagement. What we have outlined in this article is not a single transferrable model which will work for all research, all institutions and all communities. It is a flexible approach to maintaining public involvement knowledge spaces which can enable us to gain different understandings of others and, through them, of ourselves.

## Notes

1. Forced Flouridation Freedom Fighters, https://forcedfluoridationfreedomfighters.com/

2. Catalyst project, https://www.exeter.ac.uk/research/takepart/catalyst/

3.
http://clahrc-peninsula.nihr.ac.uk/penpig

4.
http://www.ecehh.org/about-us/engagement/

5.
http://www.hpru-ech.nihr.ac.uk

6.
https://www.understandingsociety.ac.uk
